# Supplementary material for: A prevalent and culturable microbiota links ecological balance to clinical stability of the human lung after transplantation
Source: Nat Commun. 2021 Apr 9;12:2126. doi: 10.1038/s41467-021-22344-4 (PMC8035266; doi:10.1038/s41467-021-22344-4)
Supplement: Supplementary file 1 — Supplementary Information [file 41467_2021_22344_MOESM1_ESM.pdf]

Supplementary Information

A prevalent and culturable microbiota links ecological balance to clinical stability of the human lung after transplantation

Short title: Microbial ecology of the transplanted human lung

Author list

Sudip Das[1], Eric Bernasconi[2*], Angela Koutsokera[2], Daniel-Adrien Wurlod[2], Vishwachi Tripathi[1], Germán Bonilla-Rosso[1], John-David Aubert[2], Marie-France Derkenne[2], Louis Mercier[2], Céline Pattaroni[2,3], Alexis Rapin[2], Christophe von Garnier[2], Benjamin J. Marsland[2,3], Philipp Engel[1*] and Laurent P. Nicod[2]

Supplementary Tables

Supplementary Table 1: BALF sample characteristics and patient demographics.

**Patients/Samples**	Total (n)	64/234
	Male / Female	25 (39.1%)/39 (60.9%)
	Age at transplant (yr)	54 (36-60)
	Sampling time point (months post-transplant)	6 (2-12)
**Type of transplant**	Bilateral lung	61 (95.3%)
	Single lung	3 (4.7%)
**Pre-transplant diagnosis**	Interstitial lung disease	17 (26.6%)
	Cystic fibrosis	14 (21.9%)
	Chronic obstructive pulmonary disease	17 (26.6%)
	Pulmonary hypertension	4 (6.3%)
	Alpha-1 antitrypsin deficiency	4 (6.3%)
	Other	8 (12.5%)
**Transbronchial biopsies**[a,b]		224 (95.7%)
	A0	182 (77.8%)
	A1	21 (9.0%)
	A2	5 (2.1%)
	B0	148 (63.2%)
	B1	39 (16.7%)
	B2	2 (0.9%)
**Immunosuppression**[a]	Tacrolimus	231 (98.7%)
	Cyclosporin	2 (0.9%)
	Everolimus	1 (0.4%)
**Antibiotics**[a,c]	PCP prophylaxis (TMP/SMX/Atovaquone)	218 (93.1%)
	Inhaled (colistin, ambisome, tobramycin)	24 (10.3%)
	Oral or IV route (miscellaneous, including azithromycin)	71 (30.3%)
**Clinical infection**[a,d]		33 (14.1%)

Abbreviations: IV=intravenous; PCP=pneumocystis, pneumonia; TMP/SMX=trimethoprim/sulfamethoxazole.

Data presented as n (% of patients or samples) or median (interquartile range)

[a] *at sampling*

[b] *grading of pulmonary allograft rejection according to guidelines of the International Society for Heart and Lung Transplantation*

[c] *antibacterial and antifungal antibiotics*

[d] *BALF positive culture requiring treatment*

Supplementary Table 2: Incidence and abundance of the 30 most prevalent and/or abundant microbiota members of the human lower respiratory tract post-transplant.

OTU_ID	Genera	I (%)	Ai (%)	Mean abundance (relative %)	Cumulative abundance (absolute, %)	Present in LuMiCol
OTU_11	*Streptococcus*	93.6	54.1	5.1	2.0	Yes
OTU_3	*Prevotella 7*	87.6	57.9	10.3	9.1	Yes
OTU_6	*Veillonella*	84.1	49.8	3.7	1.6	Yes
OTU_15	*Pseudomonas*	78.1	19.3	2.5	1.1	No
OTU_30	*Veillonella*	75.1	37.3	1.8	0.9	Yes
OTU_20	*Rothia*	70.4	21.0	1.5	0.3	Yes
OTU_8	*Alloprevotella*	67.8	27.5	3.3	1.0	No
OTU_17	*Granulicatella*	67.4	21.5	1.3	0.6	Yes
OTU_4	*Prevotella 7*	66.5	30.5	4.4	1.7	No
OTU_7	*Neisseria*	65.7	24.5	3.8	6.0	Yes
OTU_39	*Actinomyces*	64.8	25.3	1.5	1.1	Yes
OTU_1	*Pseudomonas*	62.2	10.3	7.0	7.5	Yes
OTU_41	*Gemella*	61.4	14.2	1.0	0.1	Yes
OTU_27	*Prevotella 6*	59.7	31.8	2.3	0.7	Yes
OTU_34	*Streptococcus*	59.7	18.9	1.2	0.1	Yes
OTU_107	*Veillonella*	59.2	10.3	0.8	0.3	Yes
OTU_57	*Streptococcus*	58.8	19.3	0.9	0.4	Yes
OTU_26	*Granulicatella*	57.1	9.9	1.1	0.3	No
OTU_42	*Streptococcus*	54.5	11.2	1.1	0.2	Yes
OTU_69	*Streptococcus*	52.8	12.9	0.9	0.1	Yes
OTU_21	*Porphyromonas*	52.4	16.3	2.3	1.4	No

OTU_46	*Campylobacter*	50.2	10.3	0.6	0.3	No
OTU_2	*Staphylococcus*	44.2	6.0	5.6	22.0	Yes
OTU_6768	*Streptococcus*	17.6	1.3	0.8	1.1	No
OTU_24	*Corynebacterium 1*	16.3	2.1	5.4	3.3	No
OTU_78	*Haemophilus*	16.3	1.3	3.0	1.3	No
OTU_16	*Corynebacterium 1*	6.9	1.3	4.4	9.3	No
OTU_49	*Anaerococcus*	5.2	1.3	4.4	2.9	No
OTU_234	*Anaerococcus*	4.7	1.2	1.02	0.9	No
OTU_63	*Peptoniphilus*	4.7	1.2	2.4	0.9	No

I Overall incidence of OTUs (% of samples).

Ai Incidence of OTUs at 1% relative abundance.

Supplementary Table 3: Overview of the different combinations of bacterial culture conditions used and source or references for each media. NA refers to no modifications made. Oxygen condition codes: AN- anaerobic, MI- 5% CO_2 and AE- aerobic. For full details refer to Methods.

Semi-solid media used	*Modifications/ Supplementations*	*Source*	*Media code*	*Oxygen condition*	*References*
Brewer Thioglycolate Medium	NA	Sigma-Aldrich	BT	AN	
Cooked meat media	NA	Oxoid	CM	AN, MI, AE	
Tryptic Soy media	NA	Oxoid	TS	AN, MI, AE	
Mannitol salt agar	NA	Oxoid	MS	MI, AE	
Columbia Blood agar	5% defibrinated sheep blood	Oxoid / partially in-house	CB	AN, MI, AE	
Danish blood agar	10% defibrinated sheep blood	in-house	DB	MI	doi: 10.1111/j.1469-0691.1999.tb00440.x.
Crystal Violet Erythromycin	5% defibrinated sheep blood	in-house	CV	AN	doi: 10.1128/JCM.10.6.844-849.1979.
de Man Rogosa Sharpe agar	0.1% L-cysteine, 1% D-Fructose	Oxoid / partially in-house	MR	AN, MI, AE	
GC agar	1% GCHI enrichment	Oxoid / partially in-house	GC	MI	
Chocolate agar	1% PolyViteX	biomerieux	CH	MI, AN	
Peptone Yeast extract glucose medium (modified)	DSMZ 104 medium	in-house	PYG	AN	https://www.dsmz.de/microorganisms/medium/pdf/DSMZ_Medium104.pdf
Brain Heart Infusion Tween medium	1% Tween-80	Oxoid / partially in-house	BHTW	AN	
Brain Heart Infusion medium	NA	Oxoid	BH	MI, AE	
Brucella Blood agar	NA	biomerieux	BB	AN	
Tryptone Yeast extact Glucose	Modified Engel lab		TY	AN	

Supplementary Table 4: Beta diversity summary and statistics of four Partition around medoids (PAMs) formed by samples from the human lower respiratory tract. Differences in beta diversity indices were calculate using pairwise PERMANOVA (adonis) with p-value adjusted for False Discovery rate (FDR), two-sided, 95% Confidence Interval. * *P*< 0.05, ** *P*< 0.01, *** *P*< 0.001.

Pairs	*p* value	*adj. p* value	Significance	Beta diversity measure
PAM1 vs PAM2	0.001	0.0012	**	Sorenson Index
PAM1 vs PAM3	0.001	0.0012	**	Sorenson Index
PAM1 vs PAM4	0.001	0.0012	**	Sorenson Index
PAM2 vs PAM3	0.001	0.0012	**	Sorenson Index
PAM2 vs PAM4	0.067	0.067	ns	Sorenson Index
PAM3 vs PAM4	0.001	0.0012	**	Sorenson Index
PAM1 vs PAM2	0.001	0.0012	**	Unweighted UniFrac
PAM1 vs PAM3	0.001	0.0012	**	Unweighted UniFrac
PAM1 vs PAM4	0.001	0.0012	**	Unweighted UniFrac
PAM2 vs PAM3	0.001	0.0012	**	Unweighted UniFrac
PAM2 vs PAM4	0.091	0.091	ns	Unweighted UniFrac
PAM3 vs PAM4	0.001	0.0012	**	Unweighted UniFrac
PAM1 vs PAM2	0.001	0.0015	**	Weighted UniFrac
PAM1 vs PAM3	0.001	0.0015	**	Weighted UniFrac
PAM1 vs PAM4	0.001	0.0015	**	Weighted UniFrac
PAM2 vs PAM3	0.002	0.0024	**	Weighted UniFrac
PAM2 vs PAM4	0.021	0.021	*	Weighted UniFrac
PAM3 vs PAM4	0.001	0.0015	**	Weighted UniFrac

Supplementary Table 5: Statistical evaluation of bacterial composition of PAM1. Differential abundances after enrichment analysis was calculated between each PAM and the other 3 PAMs combined, using was ART-ANOVA. Pairwise posthoc analysis was done by using Least-squares means and *p*-value correction for False Discovery Rate (FDR), two-sided, 95% Confidence Interval. * $P< 0.05$, ** $P< 0.01$, *** $P< 0.001$.

Taxonomy	Incidence (%)	Abundance (Enriched/Reduced)	Significance (Abundance)	Present in LuMiCol
Streptococcus ; OTU_11	99.1	Enriched	***	Yes
Prevotella 7 ; OTU_3	97.4	Enriched	***	Yes
Veillonella ; OTU_6	93.9	Enriched	***	Yes
Veillonella ; OTU_30	93	Enriched	NS	Yes
Granulicatella ; OTU_17	93	Enriched	**	Yes
Actinomyces ; OTU_39	89.6	Enriched	NS	Yes
Rothia ; OTU_20	88.7	Enriched	***	Yes
Granulicatella ; OTU_26	84.3	Enriched	***	No
Gemella ; OTU_41	83.5	Enriched	***	Yes
Neisseria ; OTU_7	82.6	Enriched	***	Yes
Prevotella 7 ; OTU_4	81.7	Enriched	***	No
Prevotella 6 ; OTU_27	81.7	Enriched	NS	Yes
Streptococcus ; OTU_57	81.7	Enriched	**	Yes
Pseudomonas ; OTU_15	80.9	Reduced	***	No
Alloprevotella ; OTU_8	79.1	Enriched	***	No
Streptococcus ; OTU_34	79.1	Enriched	***	Yes
Veillonella ; OTU_107	76.5	Enriched	***	Yes
Streptococcus ; OTU_69	76.5	Enriched	***	Yes
Campylobacter ; OTU_46	75.7	Enriched	***	No
Streptococcus ; OTU_42	73.9	Enriched	***	Yes
Porphyromonas ; OTU_21	67	Enriched	NS	No
Pseudomonas ; OTU_1	59.1	Reduced	***	Yes

Supplementary Table 6: Random forest confusion matrix for prediction of pneumotypes using host immune gene expression.

Pneumotypes	Balanced	*Staphylococcus*	Microbiota-depleted	*Pseudomonas*	Accuracy (%)
**Balanced**	103	0	8	1	92%
Staphylococcus	5	0	13	0	0%
**Microbiota-depleted**	13	0	61	1	81.4%
Pseudomonas	5	0	0	19	83.4%

Supplementary Table 7: Primer sequences and qPCR specifications for each primer pair used to analyze host gene expression and anelloviruses in BALF and genotyping of bacterial species.

Oligonucleotide Name /ID	Organism	Method	Sequence	Illumina Adaptor	Linker	Target	Reference / Source
nuc33F	Bacteria	PCR	GGTAGCCATCATTATTGTAGGTGT			Staphylococcal Thermonuclease (nuc)	This study
nuc432R	Bacteria	PCR	AAGTCCCTTTTCCACTAATTCCTT			Staphylococcal Thermonuclease (nuc)	This study
prLK-UV27-010 F	Bacteria	PCR	AGRGTTYGATYMTGGCTCAG			Universal 16S rRNA gene (V1-V5)	https://doi.org/10.1371/journal.pgen.1004596
prLK-UV907-011 R	Bacteria	PCR	CCGTCAATTCMTTTRAGTTT			Universal 16S rRNA gene (V1-V5)	https://doi.org/10.1371/journal.pgen.1004596
LK205	Bacteria	PCR	GCGCGCTTAAGTTAGGAGGTAGAAGATGGCAAAT			Streptococcal Pneumoylsin (ply)	Lance Keller & Jan-Willem Veening, DMF, Lausanne
LK206	Bacteria	PCR	GCGCGCTTAAGCTACTACTAGTCATTTTCTACCTTATC			Streptococcal Pneumoylsin (ply)	Lance Keller & Jan-Willem Veening, DMF, Lausanne
EB27F	Bacteria	Illumina MiSeq	**AATGATACGGCGACCACCGAGATCTACAC***TATGGTAATTCC*CAGMGTTYGATYMTGGCTCAG	**AATGATACGGCGACCACCGAGATCTACAC**	TATGGTAATTCC	Universal 16S rRNA gene (V1-V2)	-
EB338R	Bacteria	Illumina MiSeq	**CAAGCAGAAGACGGCATACGAGAT**NNNNNNNNNNNN*AGTCAGTCAGAA*GCTGCCTCCCGTAGGAGT	**CAAGCAGAAGACGGCATACGAGAT**	AGTCAGTCAGAA	Universal 16S rRNA gene (V1-V2)	-
EB926F	Bacteria	qPCR	AAACTCAAAKGAATTGACGG			Universal 16S rRNA gene (V6)	doi: 10.1016/j.mimet.2011.06.010
EB1062R	Bacteria	qPCR	CTCACRRCACGAGCTGAC			Universal 16S rRNA gene (V6)	doi: 10.1016/j.mimet.2011.06.010
NG779	Virus	qPCR	ACWKMCGAATGGCTGAGTTT			pan-Anelloviridae	10.1128/JCM.01703-07
NG780	Virus	qPCR	RGTGRCGAATGGYWGAGTTT			pan-Anelloviridae	10.1128/JCM.01703-07
NG781	Virus	qPCR	CCCKWGCCCGARTTGCCCCT			pan-Anelloviridae	10.1128/JCM.01703-07
NG782	Virus	qPCR	AYCTWGCCCGAATTGCCCCT			pan-Anelloviridae	10.1128/JCM.01703-07
NG791	Virus	qPCR	CTCACCTYSGGCWCCCGCCC			Betatorquevirus	10.1128/JCM.01703-07
NG792	Virus	qPCR	TTTATGCYGCYAGACGRAGA			Betatorquevirus	10.1128/JCM.01703-07
NG793	Virus	qPCR	TTTAYCMYGCCAGACGGAGA			Betatorquevirus	10.1128/JCM.01703-07
NG794	Virus	qPCR	TTTATGCCGCCAGACGRAGG			Betatorquevirus	10.1128/JCM.01703-07
NG795	Virus	qPCR	SGABCGAGCGCAGCGAGGAG			Gammatorquevirus	10.1128/JCM.01703-07
NG796	Virus	qPCR	GCCCGARTTGCCCCTAGACC			Gammatorquevirus	10.1128/JCM.01703-07
TTVgr1F1	Virus	qPCR	CAGTTAGTGGTGAGCCGAA			Torque Teno Virus 1	10.1128/JVI.77.4.2418–2425.2003
TTVgr1R1	Virus	qPCR	GACACAGANATTACAGCCCC			Torque Teno Virus 1	10.1128/JVI.77.4.2418–2425.2003
TTVgr1F2	Virus	qPCR	ACAGCCCCCAGCATACATCC			Torque Teno Virus 1	10.1128/JVI.77.4.2418–2425.2003
TTVgr1R2	Virus	qPCR	GTGGTGGCATAGACCGTTAG			Torque Teno Virus 1	10.1128/JVI.77.4.2418–2425.2003
TTVgr2F1	Virus	qPCR	ACANAGATGTCCCTGGAGTAGA			Torque Teno Virus 2	10.1128/JVI.77.4.2418–2425.2003
TTVgr2R1	Virus	qPCR	AGAGGCACTGATGTTTACTGGC			Torque Teno Virus 2	10.1128/JVI.77.4.2418–2425.2003
TTVgr2F2	Virus	qPCR	TGGAGTAGATCGAACGAAGA			Torque Teno Virus 2	10.1128/JVI.77.4.2418–2425.2003
TTVgr2R2	Virus	qPCR	CTGTAAATAGAGTGGGGGG			Torque Teno Virus 2	10.1128/JVI.77.4.2418–2425.2003
TTVgr3F12	Virus	qPCR	GACCARCTAGACCTGGCCAGATA			Torque Teno Virus 3	10.1128/JVI.77.4.2418–2425.2003
TTVgr3R1	Virus	qPCR	GTTTGTGGTGAGCMGAAYGG			Torque Teno Virus 3	10.1128/JVI.77.4.2418–2425.2003
TTVgr3R2	Virus	qPCR	GTACCASTKGTCTWCAAA			Torque Teno Virus 3	10.1128/JVI.77.4.2418–2425.2003
TTVgr4F1	Virus	qPCR	CCATTTTGTRCAGCCCGCCA			Torque Teno Virus 4	10.1128/JVI.77.4.2418–2425.2003

TTVgr4R1	Virus	qPCR	ACGGCATGACTTTGTGTYCTG			Torque Teno Virus 4	10.1128/JVI.77.4.2418–2425.2003
TTVgr4F2	Virus	qPCR	AGCCCGCCAATTTCTGTTC			Torque Teno Virus 4	10.1128/JVI.77.4.2418–2425.2003
TTVgr4R2	Virus	qPCR	GGAGAACAGAGCRRGGGAAT			Torque Teno Virus 4	10.1128/JVI.77.4.2418–2425.2003
TTVgr5F1	Virus	qPCR	CGTAGCCATGCTGCTGTTTG			Torque Teno Virus 5	10.1007/s705-002-8301-7
TTVgr5R1	Virus	qPCR	TCCACCATCCCCATGCCATG			Torque Teno Virus 5	10.1007/s705-002-8301-7
TTVgr5F2	Virus	qPCR	CTGTTTGTGGYTGTGGGGAT			Torque Teno Virus 5	10.1007/s705-002-8301-7
TTVgr5R2	Virus	qPCR	TCCCCATGCCATGGCAGGGC			Torque Teno Virus 5	10.1007/s705-002-8301-7

Supplementary Figures

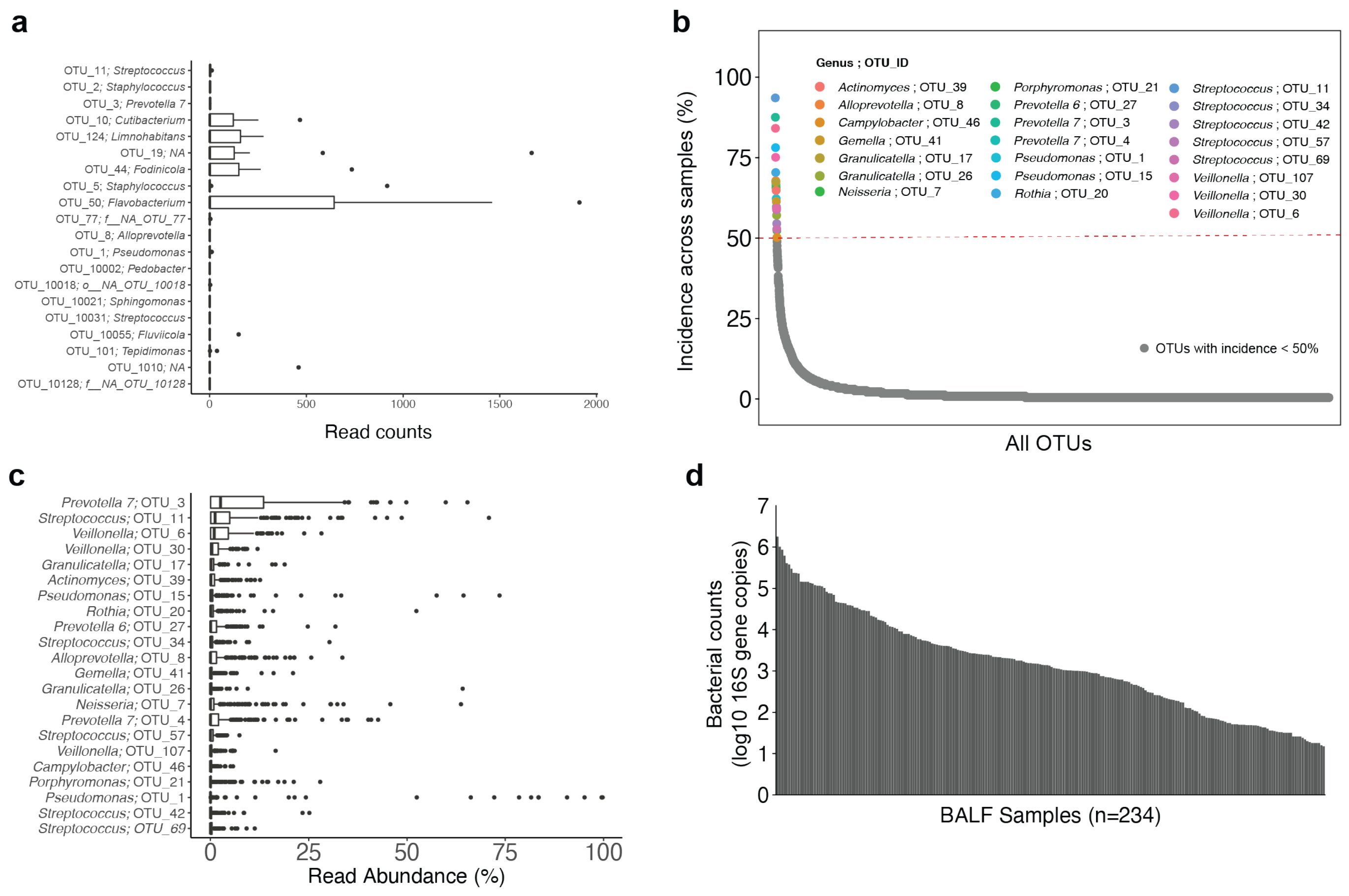

Supplementary Fig. 1. Relative abundance and prevalence analyses of OTUs detected in the negative controls as well as of the most abundant OTUs across all 234 BALF samples.

a Number of reads per BALF sample contributed by ambiguous OTUs also detected in negative control samples, which included Bronchoscope pre-wash, DNA extraction reagents, and no-template PCR reaction. **b** Incidence plot showing the frequencies of OTUs across all BALF samples (%, vertical axis) in our cohort. Dot plot show bacterial taxa (genera and OTU IDs, colored points) present in ≥50% of BALF samples (red dotted line), while grey points show OTUs with incidences ≤ 50%. **c** Relative read abundances (%) of the most abundant OTUs (genera; OTU IDs) across all BALF samples. Box plots show median (middle line), 25th, 75th percentile (box) and 5th and 95th percentile (whiskers) as well as outliers (single points). **d** Barplot showing the distribution of bacterial counts (log_{10} 16S gene copies) determined by qPCR on the 16S rRNA gene (vertical axis) across all samples (n= 234, horizontal axis). All box plots show median (middle line), 25th, 75th percentile (box) and 5th and 95th percentile (whiskers) as well as outliers (single points).

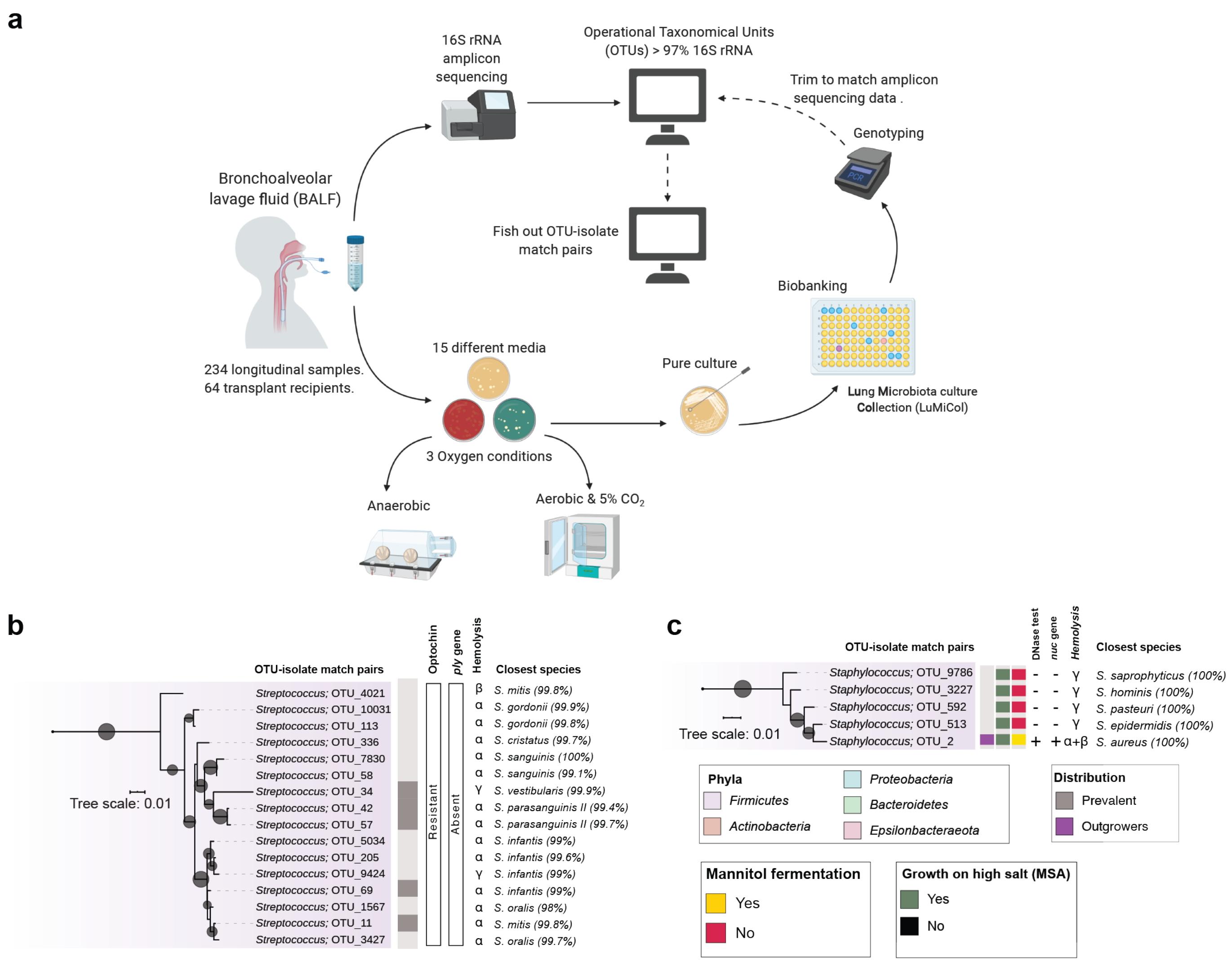

Supplementary Fig. 2. Workflow of our combined approach of BALF amplicon sequencing and bacterial culture to deduce the microbial ecology of deep lung microbiota. a Amplicon sequencing of the 16S rRNA gene was carried out for 234 bronchoalveolar lavage fluid (BALF) samples from 64 recipients post-lung transplant. The resulting reads were clustered into operational taxonomic units (OTUs) to determine the community composition of each sample. For a subset of the samples, bacteria were cultured on 15 different media under 3 oxygen conditions. Single colonies were picked, genotyped, and arrayed into a bacterial strain collection referred to as LuMiCol. 16S rRNA gene sequences of these isolates were included into the community analysis based on the culture-independent 16S rRNA gene amplicon sequences to identify which isolate belongs to which OTU (OTU-isolate matching pairs). Original graphical art "Created using BioRender.com". **b,** Phenotypic tests to differentiate bacterial species. Taxa are denoted by genera and OTU IDs, and phyla are shown with colored highlights and also designated as prevalent (grey rectangle)

or opportunists (magenta rectangle) of the lower respiratory community. Streptococci were confirmed by their characteristic hemolysis. Viridans streptococci were differentiated from pneumococcus by optochin resistance test and the presence of *ply* gene encoding pneumolysin toxin. **c** *Staphylococcus aureus* was differentiated from other staphylococci by its ability to grow in high salt concentration and fermentation of mannitol (Mannitol Salt Agar), characteristic hemolysis, presence of *nuc* gene encoding for staphylococcal thermonuclease and extracellular DNase activity assay.

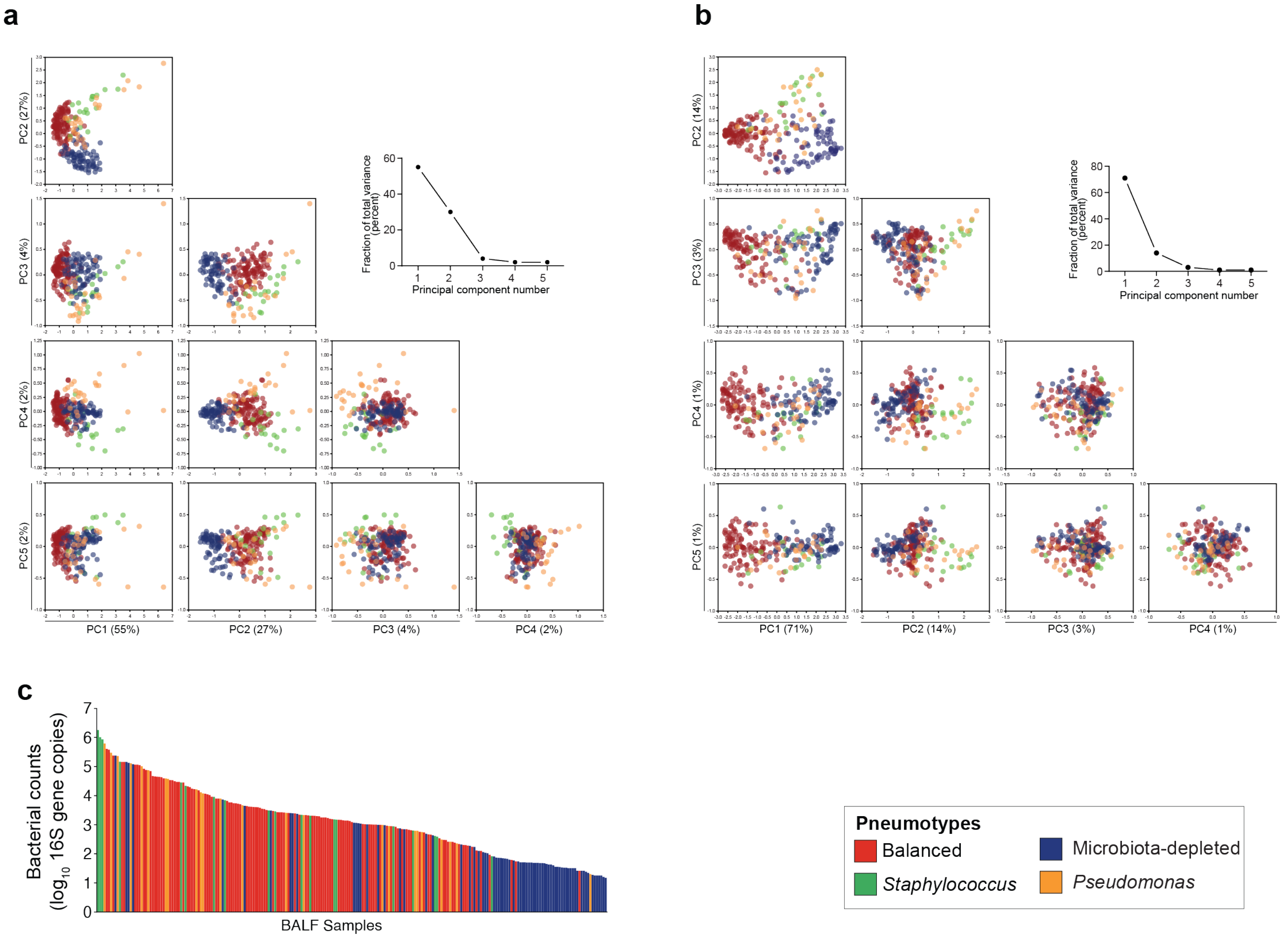

Supplementary Fig. 3. Principal component analysis based on the bacterial community compositions and bacterial loads of the 234 BALF samples. a, b Clustering of samples based on genus level (**a**) and OTU level (**b**) along principal components 1 to 5. Samples are coloured according to PAM designation. Line graph shows total variance explained per principal component. **c** Barplot showing the distribution of bacterial counts (log_{10} 16S gene copies) determined by qPCR on the 16S rRNA gene (vertical axis) across all samples (n= 234, horizontal axis). Samples are sorted according to load and colors correspond to PAM designation. PAM1, PAM2, PAM3, and PAM4 are coloured in red, green, blue, and orange, respectively. For details on input and output parameters and additional dataset refer to Supplementary Data.

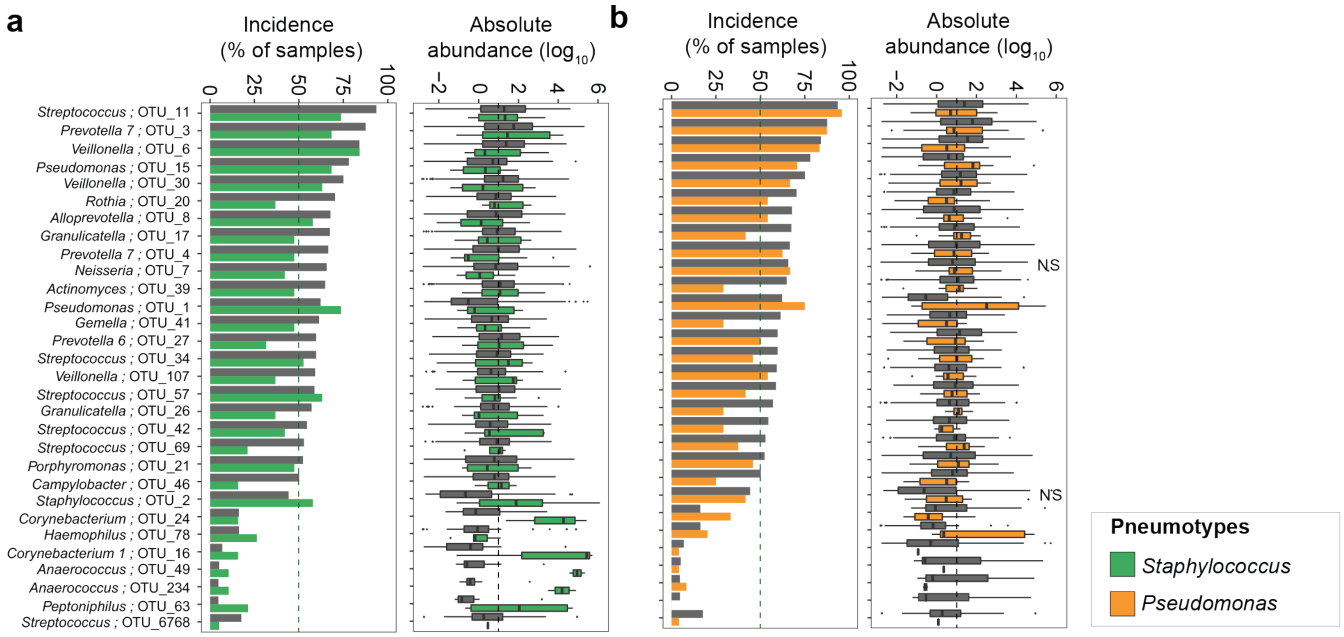

Supplementary Fig. 4. Prevalence (i.e % of samples with a specific OTU) and absolute abundance across all samples of the 30 most dominant bacterial community members (i.e. OTUs) in each PAM2 and PAM4 as compared to the other 3 PAMs.

Green and orange graphs correspond to values for PAM2 (**a**) and PAM4 (**b**), respectively, while grey graphs correspond to values for other 3 PAMs excluding the one in comparison. Incidence of 50% is indicated by the green dotted line. Enrichment analysis was performed on both incidences and differential abundances after enrichment analysis was calculated between each PAM and the other 3 PAMs combined, using ART-ANOVA. Box plots show median (middle line), 25th, 75th percentile (box) and 5th and 95th percentile (whiskers) as well as outliers (single points). Posthoc analyses (95% Confidence Interval) were done by using least-squares means (ART-ANOVA) with False Discovery Rate (FDR). * *P*< 0.05, ** *P*< 0.01, *** *P*< 0.001, NS= not significant.

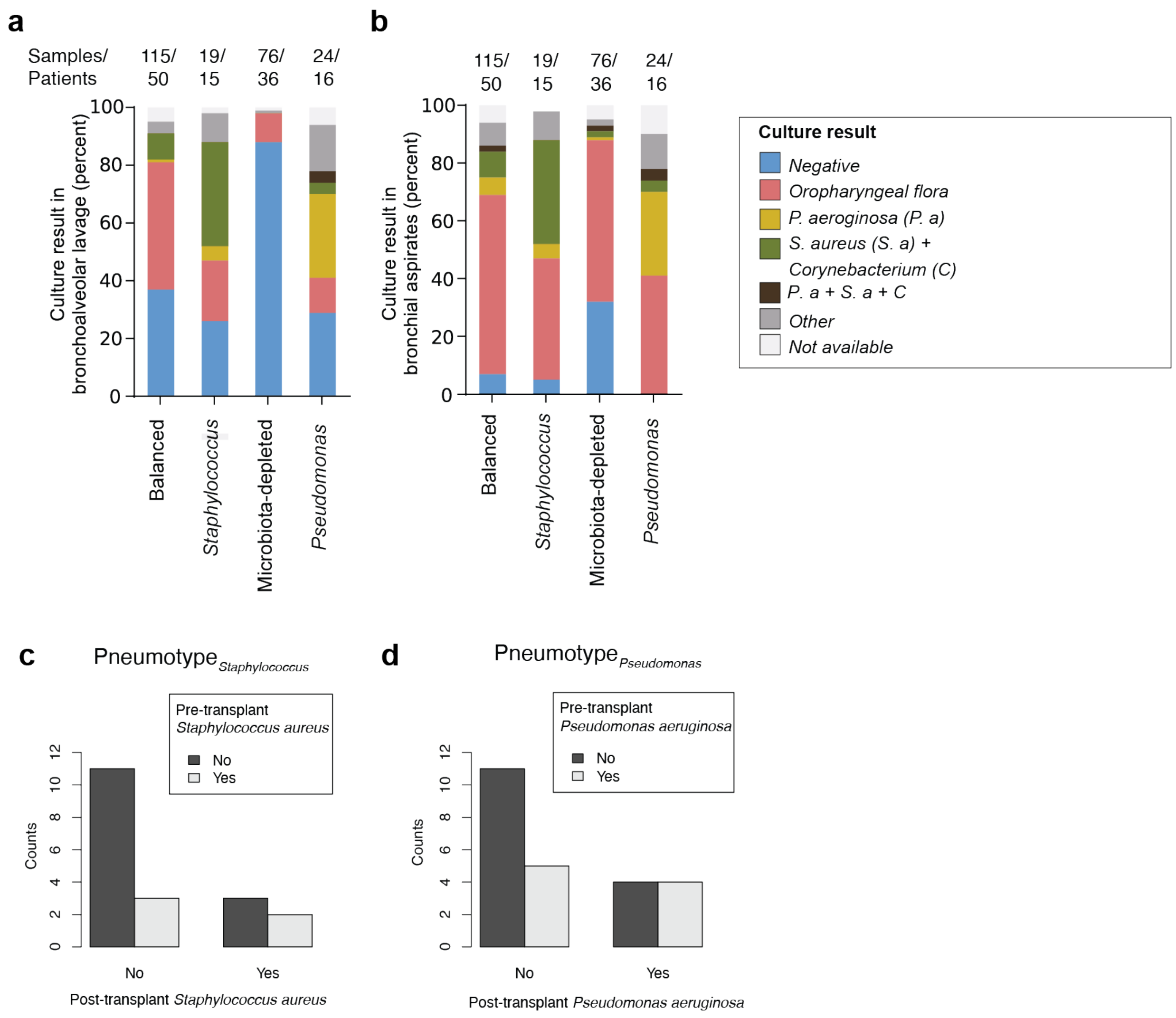

Supplementary Fig. 5. Comparison of culturing results from BALF and Bronchial aspirate (BAs) samples belonging to different pneumotypes (as based on BALF community analysis) confirms the presence of distinct microbial communities. **a, b** Stacked bar plots showing relative percentage of specific taxonomic groups (colored bars) isolated from paired Bronchial aspirates (BA) and Bronchoalveolar lavage fluid (BALF) samples, plotted according to the Pneumotype designation of each sample pair. **c, d** Count comparison of *Staphylococcus aureus* (Chi-square test, χ^2 =0.047, *p*= 0.82) and *Pseudomonas aeruginosa* (Chi-square test, χ^2 =0.2, *p*= 0.65) colonisation in Bronchial aspirates pre-transplant (bar colors) and BAL post-transplant (x-axis) in association with Pneumotype$_{Staphylococcus}$ and Pneumotype$_{Pseudomonas}$ respectively.

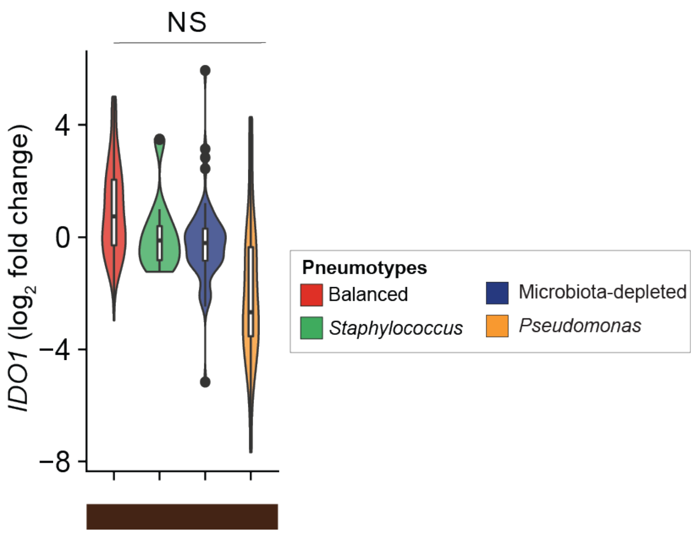

Supplementary Fig. 6. Gene expression differences of peripheral immune tolerance gene IDO1 across the four pneumotypes.

Violin plots show distribution of *IDO1* expression (log_2 fold) across the four pneumotypes (n=229, ANOVA, $F(3, 225)= 2.28$, two-sided, $P= 7.9x10^{-2}$). The functional category is shown at the bottom of the plot according to the color scheme used in Fig. 4a. All box plots including insets show median (middle line), 25th, 75th percentile (box) and 5th and 95th percentile (whiskers) as well as outliers (single points). Posthoc analyses (95% Confidence Interval) were done by using Tukey's test (ANOVA) or Dunn's test (Kruskal test) with False Discovery Rate (FDR). * $P< 0.05$, ** $P< 0.01$, *** $P< 0.001$, NS= not significant.

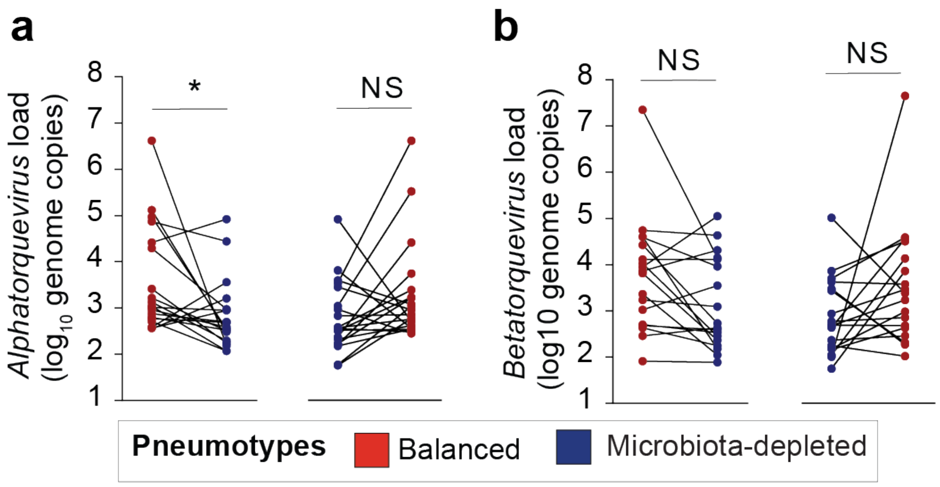

Supplementary Fig. 7. Burden of major anellovirus genera in BALF differ between pneumotypes

Intra-individual analysis of *Alpha-* and *Betatorquevirus* loads (log_{10} genome copies per ml BALF) for the samples transitioning from $Pneumotype_{Balanced}$ (Red) to $Pneumotype_{MD}$ (Blue) (*Alphatorquevirus:* n=19, Wilcoxon test, two-sided, paired, *P*= 4.4×10^{-2}, *Betatorquevirus:* n=18, Wilcoxon test, two-sided, paired, *P*= 6.6×10^{-2}), and from $Pneumotype_{MD}$ to $Pneumotype_{Balanced}$ (*Alphatorquevirus:* n=20, Wilcoxon test, two-sided, paired, *P*= 2.1×10^{-1}, *Betatorquevirus:* n=18, Wilcoxon test, two-sided, paired, *P*= 1.0×10^{-1}). Paired data (joined by black lines) presented here are viral genome copies (log_{10}, points). Posthoc analyses (95% Confidence Interval) were done by using Tukey's test (ANOVA) or Dunn's test (Kruskal test) with False Discovery Rate (FDR). * *P*< 0.05, ** *P*< 0.01, *** *P*< 0.001, NS= not significant.

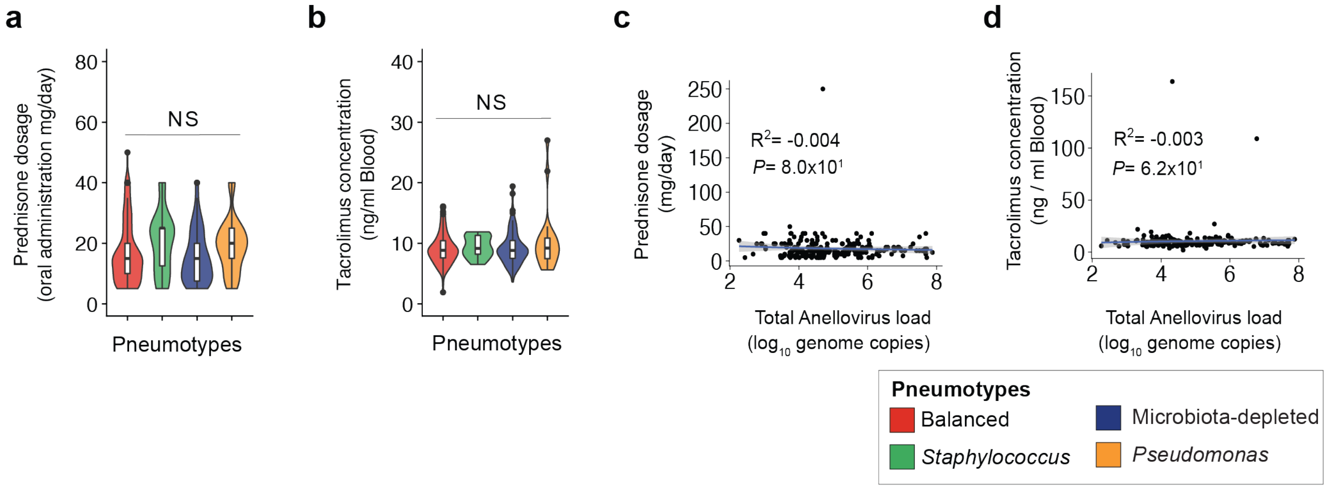

Supplementary Fig. 8. Association of immunosuppresant drugs with pneumotypes and anellovirus load in BALF. a, b Violin plots showing distribution of immunosuppresants levels: Prednisone dosage (mg/day, n= 225, ANOVA, two-sided, $F(3, 221) = 0.38$, $P= 7.68\times10^{1}$) and Tacrolimus concentration in blood (ng/ml, n= 230, ANOVA, two-sided, $F(3, 226) = 0.46$, $P= 7.08\times10^{1}$) in BALF samples from four pneumotypes (plot colors). Box plots insets show median (middle line), 25th, 75th percentile (box) and 5th and 95th percentile (whiskers) as well as outliers (single points). Posthoc analyses were done by using Tukey's test (ANOVA) or Dunn's test with *p*-value correction for False Discovery Rate (FDR), 95% Confidence Interval. * $P< 0.05$, ** $P< 0.01$, *** $P< 0.001$.**c, d** Scatter plots show correlation between levels of immunosuppresants: Prednisone dosage (mg/day, n=215, two-sided, $F(1, 214) = 0.06$, $P= 8.0\times10^{1}$) and Tacrolimus concentration in blood (ng/ml, n=221, two-sided, $F(1, 219) = 0.012$, $P= 9.1\times10^{1}$) and total anellovirus burden ($\log_{10}$ genome copies per ml BALF for each sample. Linear regression is shown by the blue line with grey shaded area showing 95% confidence interval Coefficient of correlation; adjusted $R^2 = -0.0043$ and -0.0043, respectively.

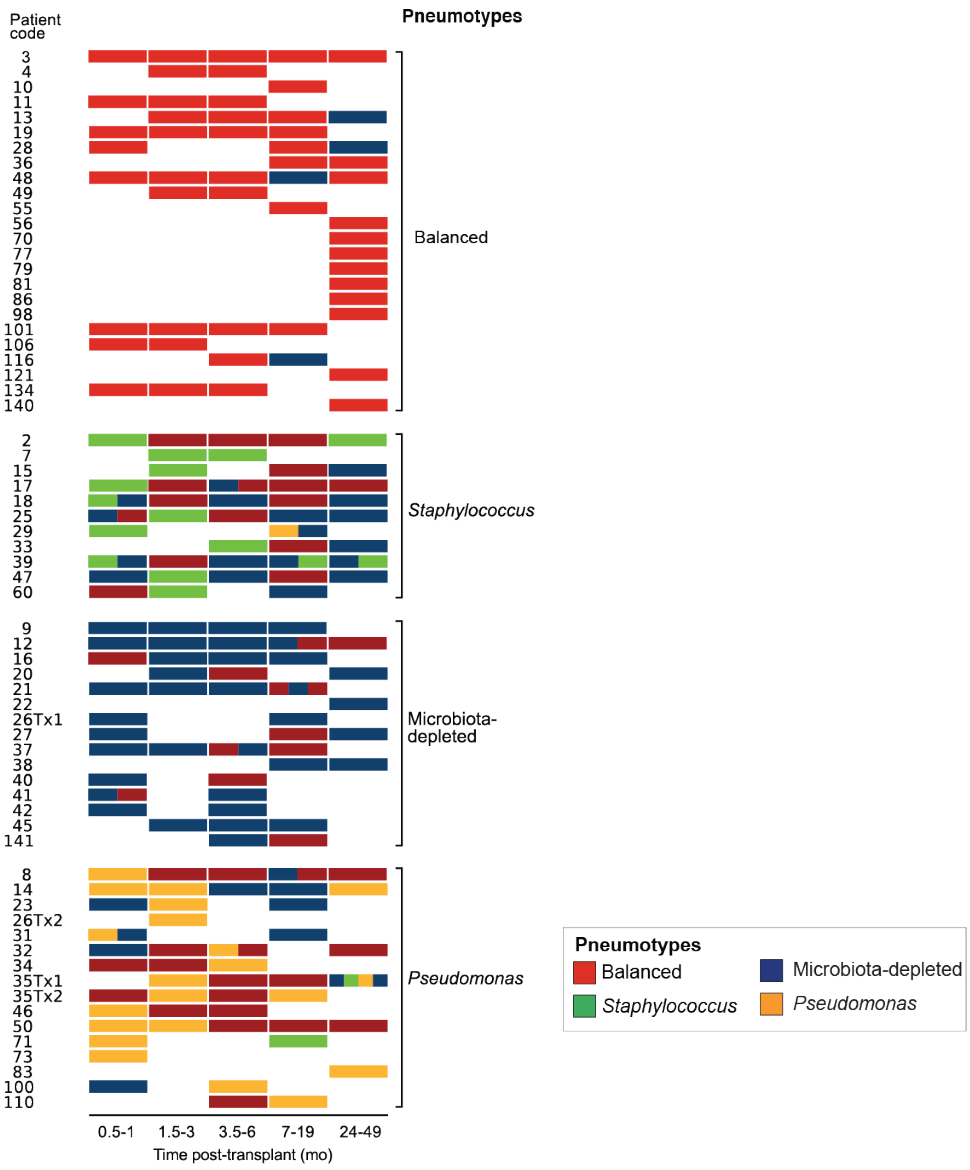

Supplementary Fig. 9. Longitudinal analysis of lung microbiota post-transplant to investigate pneumotype transitions.

Longitudinal BALF sampling from patients with given IDs and integrated pneumotype information across time (months post-transplant).
