# Supplementary material for: A prevalent and culturable microbiota links ecological balance to clinical stability of the human lung after transplantation
Source: Nat Commun. 2021 Apr 9;12:2126. doi: 10.1038/s41467-021-22344-4 (PMC8035266; doi:10.1038/s41467-021-22344-4)
Supplement: Supplementary file 3 — Description of Additional Supplementary Files [file 41467_2021_22344_MOESM3_ESM.docx]

**Description of Additional Supplementary Files**

**A prevalent and culturable microbiota links ecological balance to clinical stability of the human lung after transplantation**

Short title: Microbial ecology of the transplanted human lung

**Author list**

Sudip Das^1^, Eric Bernasconi^2^*, Angela Koutsokera^2^, Daniel-Adrien Wurlod^2^, Vishwachi Tripathi^1^, Germán Bonilla-Rosso^1^, John-David Aubert^2^, Marie-France Derkenne^2^, Louis Mercier^2^, Céline Pattaroni^2,3^, Alexis Rapin^2^, Christophe von Garnier^2^, Benjamin J. Marsland^2,3^, Philipp Engel^1^* and Laurent P. Nicod^2^

**File Name:** Supplementary_Data_1

**Description:**Table of 16S rRNA amplicon sequencing information, such as OTU relative abundance and taxonomy, by sample, without negative controls.

**File Name:** Supplementary_Data_2

**Description:**Table of 16S rRNA amplicon sequencing information, such as OTU relative abundance and taxonomy, for each negative control.

**File Name:** Supplementary_Data_3

**Description:**Detailed table showing frequency of all OTUs detected across the 234 BALF samples in terms of their abundance and prevalence.

**File Name:** Supplementary_Data_4

**Description:**List of Lung microbiota culture collection (LuMiCol) isolates with detailed information about sample number, culture conditions, taxonomy. This is a non-curated list containing about 300 isolates of which 215 were used for analysis.

**File Name:** Supplementary_Data_5

**Description:**OTU-isolate match summary listing the isolates that match OTUs in their 16S rRNA gene sequence, and information about the number of representative isolates in LuMiCol, with at least one representative isolate name, prevalence, and preference in terms of oxygen condition and media.

**File Name:** Supplementary_Data_6

**Description:**Detailed input and output files from unsupervised machine learning approach used by Genocrunch to determine Partition around medoids (PAMs) i.e. pneumotypes. The zip file includes parameters files: json and text files and Silhouette plots.

**File Name: Supplementary_Data_7**

**Description:**Detailed metadata table associated with all patients and samples.
