# Supplementary material for: A prevalent and culturable microbiota links ecological balance to clinical stability of the human lung after transplantation
Source: Nat Commun. 2021 Apr 9;12:2126. doi: 10.1038/s41467-021-22344-4 (PMC8035266; doi:10.1038/s41467-021-22344-4)
Supplement: Supplementary file 9 — Supplementary Data 6 [file 41467_2021_22344_MOESM9_ESM.zip › Supplementary_Data_6/output/clustering/clustering_level_5.pdf]

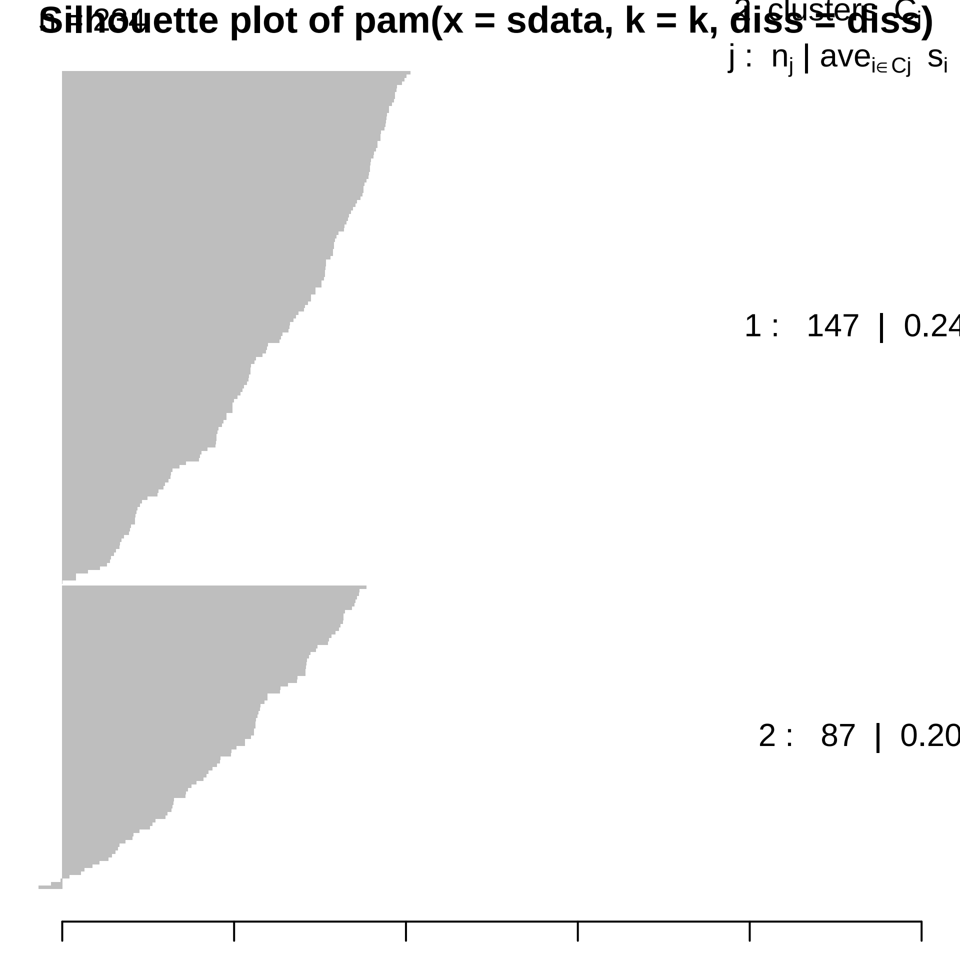
Silhouette plot of pam(x = sdata, k = k, diss = diss)
n = 234
2 clusters Cj
j : nj | avei∈Cj si
1 : 147 | 0.24
2 : 87 | 0.20
