# Supplementary material for: A prevalent and culturable microbiota links ecological balance to clinical stability of the human lung after transplantation
Source: Nat Commun. 2021 Apr 9;12:2126. doi: 10.1038/s41467-021-22344-4 (PMC8035266; doi:10.1038/s41467-021-22344-4)
Supplement: Supplementary file 9 — Supplementary Data 6 [file 41467_2021_22344_MOESM9_ESM.zip › Supplementary_Data_6/output/clustering/clustering_level_2.pdf]

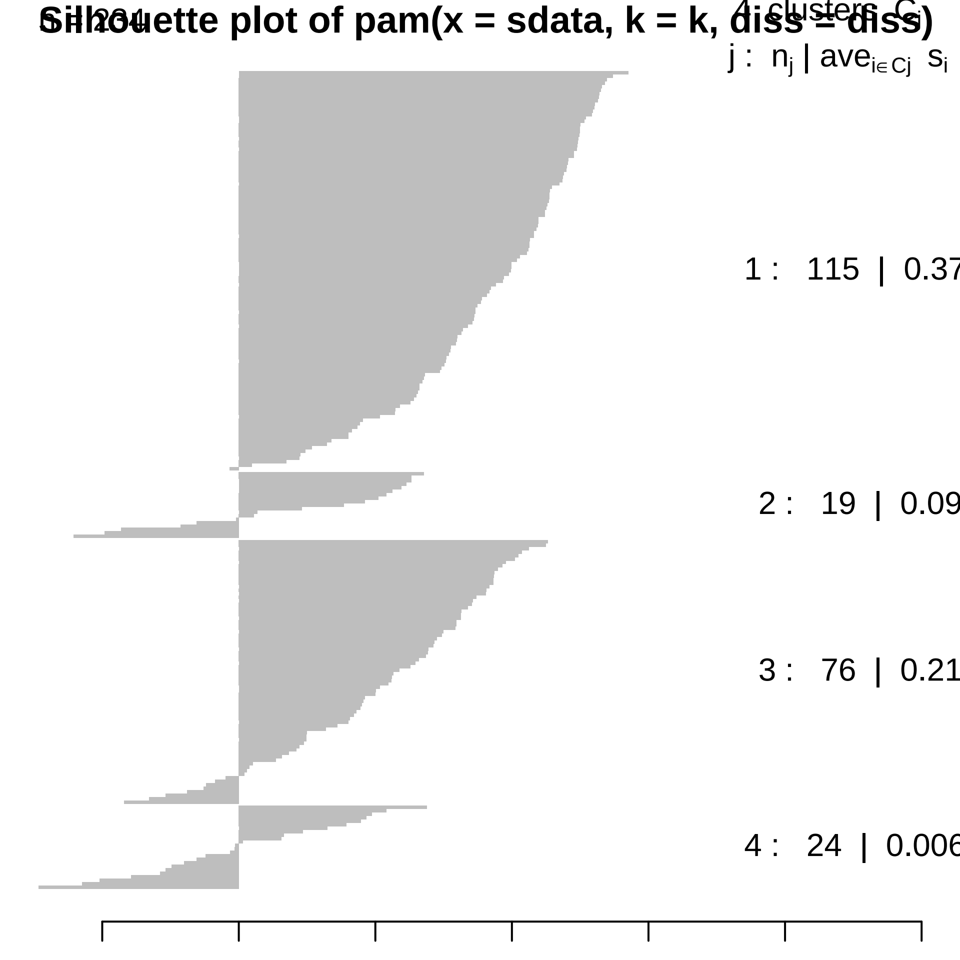
Silhouette plot of pam(x = sdata, k = k, diss = diss)
n = 234
4 clusters C_j
j : n_j | $ave_{i \in Cj}$ s_i
1 : 115 | 0.37
2 : 19 | 0.09
3 : 76 | 0.21
4 : 24 | 0.006
