# Supplementary figures and images for: A prevalent and culturable microbiota links ecological balance to clinical stability of the human lung after transplantation
Source: Nat Commun. 2021 Apr 9;12:2126. doi: 10.1038/s41467-021-22344-4 (PMC8035266; doi:10.1038/s41467-021-22344-4)

Individuals factor map (PCA)

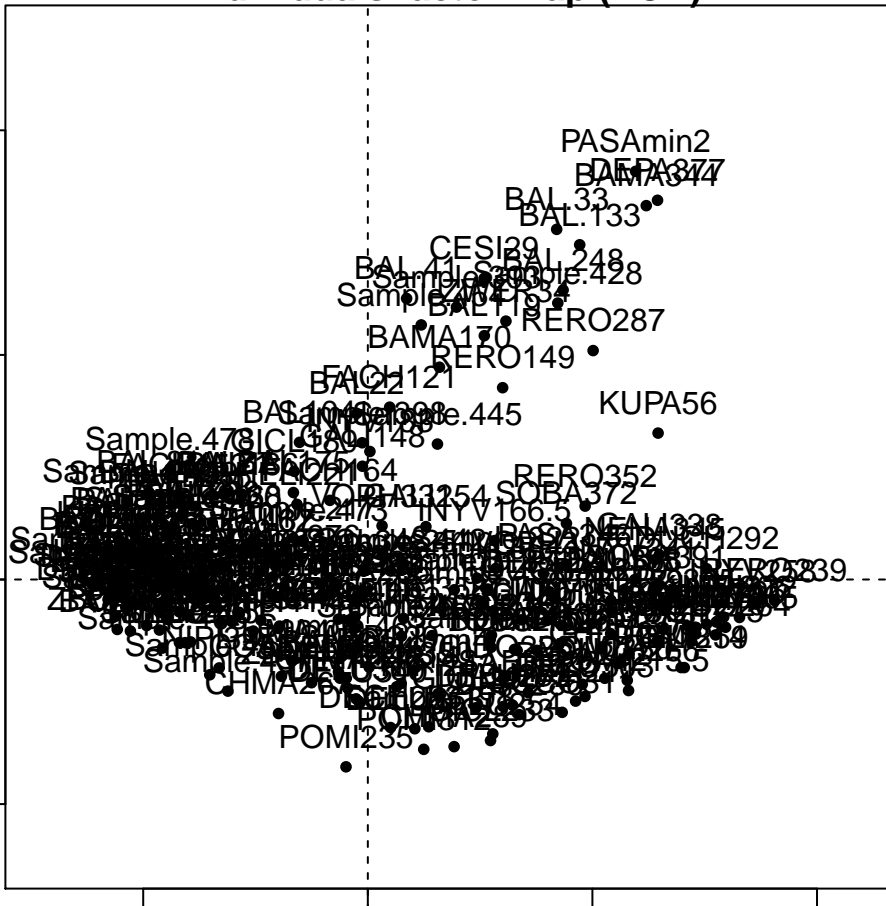

Supplement: Supplementary file 9 — Supplementary Data 6 [file 41467_2021_22344_MOESM9_ESM.zip › Supplementary_Data_6/output/pcoa/pcoa_level_5.pdf]

Individuals factor map (PCA)

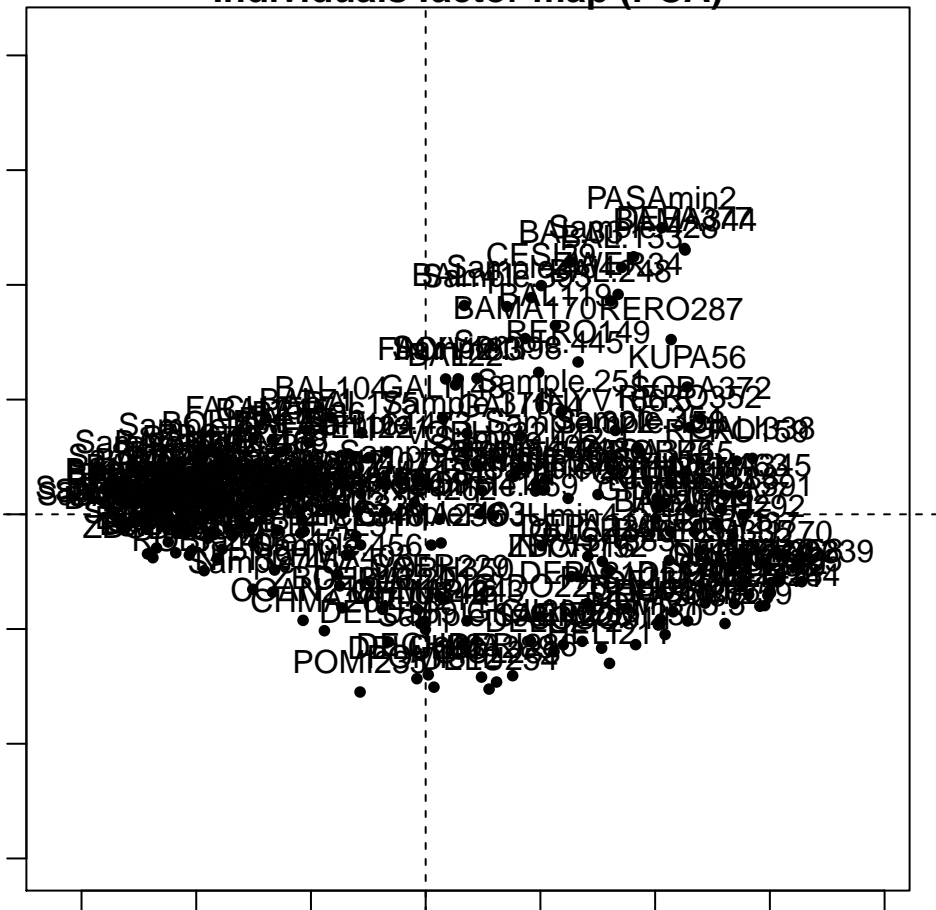

Supplement: Supplementary file 9 — Supplementary Data 6 [file 41467_2021_22344_MOESM9_ESM.zip › Supplementary_Data_6/output/pcoa/pcoa_level_7.pdf]

Individuals factor map (PCA)

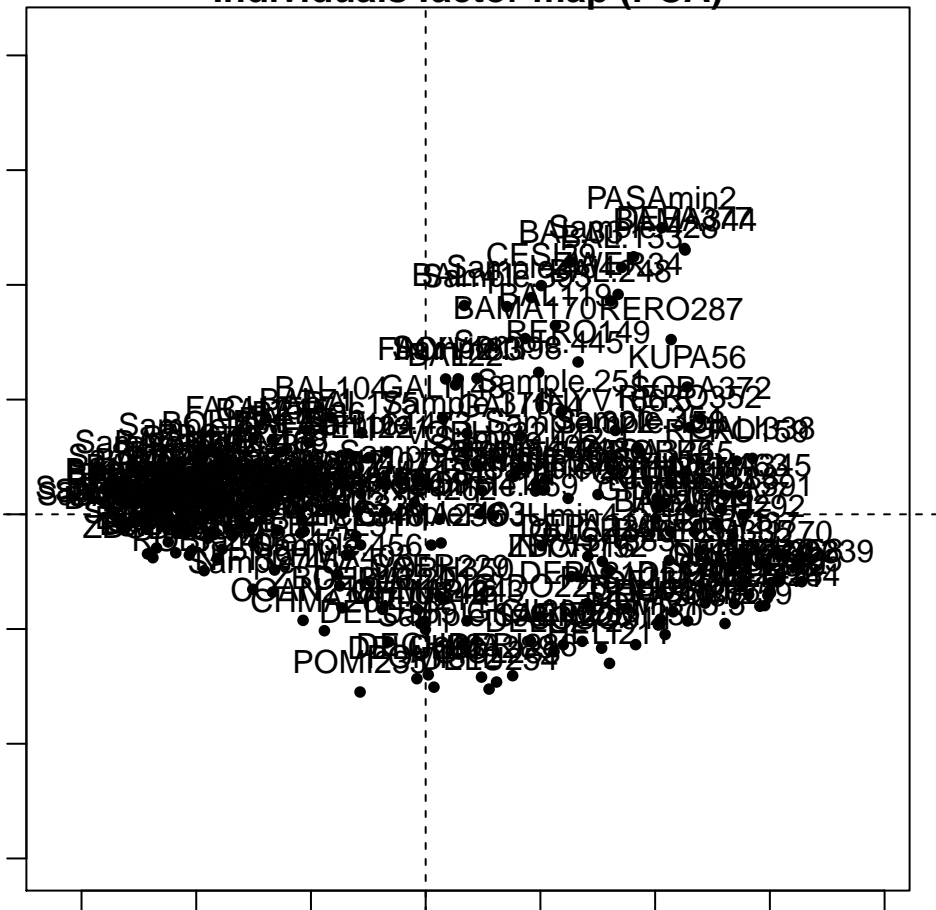

Supplement: Supplementary file 9 — Supplementary Data 6 [file 41467_2021_22344_MOESM9_ESM.zip › Supplementary_Data_6/output/pcoa/pcoa_level_6.pdf]
