# Supplementary material for: A prevalent and culturable microbiota links ecological balance to clinical stability of the human lung after transplantation
Source: Nat Commun. 2021 Apr 9;12:2126. doi: 10.1038/s41467-021-22344-4 (PMC8035266; doi:10.1038/s41467-021-22344-4)
Supplement: Supplementary file 11 — Reporting Summary [file 41467_2021_22344_MOESM11_ESM.docx]

1

nature research | reporting summary

*April 2020*


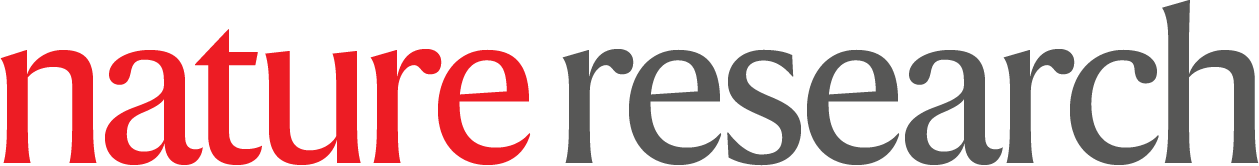
Corresponding author(s): Eric Bernasconi, Philipp Engel

Last updated by author(s): Feb 26, 2021

Reporting Summary

Nature Research wishes to improve the reproducibility of the work that we publish. This form provides structure for consistency and transparency in reporting. For further information on Nature Research policies, see our Editorial Policies and the Editorial Policy Checklist.

Please do not complete any field with "not applicable" or n/a. Refer to the help text for what text to use if an item is not relevant to your study. For final submission: please carefully check your responses for accuracy; you will not be able to make changes later.

## Statistics

For all statistical analyses, confirm that the following items are present in the figure legend, table legend, main text, or Methods section. n/a Confirmed


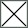

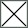

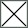

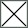


The exact sample size (*n*) for each experimental group/condition, given as a discrete number and unit of measurement

A statement on whether measurements were taken from distinct samples or whether the same sample was measured repeatedly The statistical test(s) used AND whether they are one- or two-sided

*Only common tests should be described solely by name; describe more complex techniques in the Methods section.*

A description of all covariates tested

A description of any assumptions or corrections, such as tests of normality and adjustment for multiple comparisons

A full description of the statistical parameters including central tendency (e.g. means) or other basic estimates (e.g. regression coefficient) AND variation (e.g. standard deviation) or associated estimates of uncertainty (e.g. confidence intervals)

For null hypothesis testing, the test statistic (e.g. *F*, *t*, *r*) with confidence intervals, effect sizes, degrees of freedom and *P* value noted

*Give P values as exact values whenever suitable.*

For Bayesian analysis, information on the choice of priors and Markov chain Monte Carlo settings

For hierarchical and complex designs, identification of the appropriate level for tests and full reporting of outcomes Estimates of effect sizes (e.g. Cohen's *d*, Pearson's *r*), indicating how they were calculated

*Our web collection on statistics for biologists contains articles on many of the points above.*

## Software and code

Policy information about availability of computer code Data collection

No software was used for data collection

Data analysis

Code and version used to analyse the data have been deposited in GitHub (https://github.com/sudu87/Microbial-ecology-of-the- transplanted-human-lung). A statement on custom code is provided in the manuscript under a separate 'code availability' section.

For manuscripts utilizing custom algorithms or software that are central to the research but not yet described in published literature, software must be made available to editors and reviewers. We strongly encourage code deposition in a community repository (e.g. GitHub). See the Nature Research guidelines for submitting code & software for further information.

## Data

Policy information about availability of data

All manuscripts must include a data availability statement. This statement should provide the following information, where applicable:

- Accession codes, unique identifiers, or web links for publicly available datasets
- A list of figures that have associated raw data
- A description of any restrictions on data availability

We have deposited the raw data from all samples used in the study to Short Read Archive, NCBI under the BioProject PRJNA632552 and BioSample accession SAMN 14911405. All codes are available for access on https://github.com/sudu87 /Microbial-ecology-of-the-transplanted-human-lung, and on the following cloud drive: https://drive.switch.ch/index.php/s/hch0EoASQyjB PRS. The content is as follows: Das_et_al_2020_analysis_pipeline_l: Sequencing analysis pipeline using python (QIIME) and bash (vsearch, FastXToolkit, SINA, FastTree); and code from raw data processing, merging cultured sequences (LuMiCol), OTU picking and phylogeny. Das_et_al_2020_analysis_pipeline_2: R markdown of BALF community analysis with starting OTUs from pipeline 1 using phyloseq, ampvis2 and vegan; and Random Forest algorithms, Markov chain analysis and ail statistical analysis and visualization plots. AII_BAL_samples_raw_fastqc: FastQC reports for raw sequencing data after merging ail samples. AII_BAL_samples_processed_fastqc: FastQC reports for trimmed and curated merged data.

2

nature research | reporting summary

*April 2020*

# Field-specific reporting

Please select the one below that is the best fit for your research. If you are not sure, read the appropriate sections before making your selection.

Life sciences Behavioural & social sciences Ecological, evolutionary & environmental sciences

For a reference copy of the document with all sections, see nature.com/documents/nr-reporting-summary-flat.pdf

# Life sciences study design

All studies must disclose on these points even when the disclosure is negative. Sample size

No sample size calculations were made for this observational, single-centre, longitudinal study in which we prospectively collected 234 bronchoalveolar lavage samples (n=1-12 per recipient, mean 3.7) from a cohort of 64 consecutive lung transplant recipients. In particular, the relatively small proportion of samples with a pneumotype of Pseudomonas or Staphylococcus, as well as the small proportion of patients developing CLAD would have required a very long sampling time to achieve sufficient statistical power. We have extended our sample collection from October 2012 to May 2018, including samples obtained between 2 weeks and 49 months after transplantation, in order to study the temporal dynamics of lung microbiota profiles in as much detail as possible, given the limitations imposed by the invasive nature of bronchoscopy. In addition, our data show that they were sufficient to train predictive random forest models, thanks to iterations.

Data exclusions

All available clinical samples obtained from patients who provided written consent were tested without exclusion. In 16S rRNA sequencing data, we excluded OTUs contributing to reads in 11 negative control samples, which included bronchoscope pre-wash, DNA extraction reagents and no-template PCR reaction.

Replication

Sequencing of 16S rRNA amplicon by Illumina Miseq was performed in three different runs, with no association between the runs and the four microbiota profiles identified in this study. The reproducibility of qPCR analysis for host gene expression, copy numbers of 16S RNA gene, or copy numbers of anellovirus genome, was ensured by the use of purified standards.

Randomization

The design of this prospective observational study did not require randomization of samples. We detected and characterized differences in bacterial community composition between all samples, using k-medoid-based unsupervised machine learning based on pairwise Bray-Curtis dissimilarity. The program segregates samples while optimizing the number of clusters based on the average silhouette width as indicated in Methods.

Blinding

Given the study design, investigators were blinded during sample collection, library preparation, post-sequencing community analysis, up to cluster identification.

# Reporting for specific materials, systems and methods

We require information from authors about some types of materials, experimental systems and methods used in many studies. Here, indicate whether each material, system or method listed is relevant to your study. If you are not sure if a list item applies to your research, read the appropriate section before selecting a response.

### Materials & experimental systems Methods


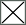

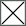

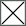

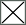

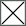


n/a Involved in the study Antibodies Eukaryotic cell lines

Palaeontology and archaeology Animals and other organisms Human research participants Clinical data

Dual use research of concern


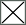

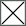

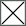


n/a Involved in the study

ChIP-seq

Flow cytometry

MRI-based neuroimaging

Human research participants

Policy information about studies involving human research participants Population characteristics

The covariate-relevant population characteristics have been specified in Table S1. They include gender, age, at transplant, sampling time point post-transplant, type of transplant, pre-transplant diagnosis, transbronchial biopsy scoring, immunosuppressants, antibiotics, and clinical infection.

Recruitment

For this prospective study, we collected 234 bronchoalveolar lavage samples from a cohort of 64 consecutive lung transplant recipients at our centre. Collection took place from October 2012 to May 2018, and samples were obtained between 2 weeks and 49 months after transplantation, during routine surveillance or clinically indicated bronchoscopies.

Ethics oversight

The study was approved by the ethics committee, “Commission cantonale (VD) d'éthique de la recherche sur l'être humain –

3

nature research | reporting summary

*April 2020*

Ethics oversight

CER-VD”, Lausanne, Switzerland, protocol number 2018-01818.

Note that full information on the approval of the study protocol must also be provided in the manuscript.

## Clinical data

Policy information about clinical studies

All manuscripts should comply with the ICMJE guidelines for publication of clinical research and a completed CONSORT checklist must be included with all submissions.

Clinical trial registration

This study is not a phase 2 or 3 randomized controlled trial.

Study protocol

The protocol of this translational research study is part of the document number 2018-01818 approved by "Commission cantonale (VD) d'éthique de la recherche sur l'être humain – CER-VD", affiliated to the Swiss Association of Research Ethics Committees, Swissethics.

Data collection

For this monocentric study, patients were recruited and their samples were collected prospectively between October 2012 and May 2018.

Outcomes

This exploratory, non-interventional study did not have primary and secondary outcomes per se. However, an important clinical measure was Chronic Lung Allograft dysfunction (CLAD), considered as described below and in the manuscript:

CLAD was defined as a loss of more than 20% of the expiratory volume in 1 second (FEV1) of the mean of the two best values (i.e. the baseline FEV1) since transplantation, without other obvious cause and without reversibility, in accordance with the diagnostic criteria specified by the Pulmonary Council of the International Society for Heart and Lung Transplantation (Ref. 54).


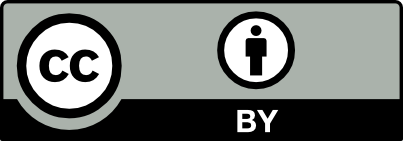
This checklist template is licensed under a Creative Commons Attribution 4.0 International License, which permits use, sharing, adaptation, distribution and reproduction in any medium or format, as long as you give appropriate credit to the original author(s) and the source, provide a link to the Creative Commons license, and indicate if changes were made. The images or other third party material in this article are included in the article's Creative Commons license, unless indicated otherwise in a credit line to the material. If material is not included in the article's Creative Commons license and your intended use is not permitted by statutory regulation or exceeds the permitted use, you will need to obtain permission directly from the copyright holder. To view a copy of this license, visit <http://creativecommons.org/licenses/by/4.0/>
